# A novel contrast-induced acute kidney injury mouse model based on low-osmolar contrast medium

**DOI:** 10.1080/0886022X.2022.2108449

**Published:** 2022-08-08

**Authors:** Jiajia Wu, Jianxiao Shen, Wanpeng Wang, Na Jiang, Haijiao Jin, Xiajing Che, Zhaohui Ni, Yan Fang, Shan Mou

**Affiliations:** a Department of Nephrology, Renji Hospital, School of Medicine, Shanghai Jiao Tong University, Shanghai, China; b Department of Nephrology, Lianshui People’s Hospital, Lianshui, China

**Keywords:** Contrast-induced acute kidney injury, mouse model, low-osmolar contrast medium

## Abstract

The contrast-induced acute kidney injury (CI-AKI) has been becoming the third common cause of hospital-acquired acute kidney injury. An ideal animal model is essential for understanding the pathophysiology of CI-AKI. Previous CI-AKI studies were mostly performed on rats with high-osmolar contrast medium (HOCM), which is unsuitable for transgenic researches. This study provides a novel, efficient and reproducible CI-AKI model which was developed in mouse by administrating a low-osmolar contrast medium (LOCM). First of all, we applied the frequently used pretreatments (uninephrectomy and water deprivation), which combined with HOCM on rats could induce CI-AKI, on mice with LOCM. Secondly, we attempted to find a novel pretreatment suitable for mouse and LOCM by combining two classic pretreatments(uninephrectomy, water deprivation and furosemide administration). Finally, we evaluate the kidney damage of the novel model. We found that this mouse model possessed a significant reduction in renal function, severe renal tissue damage, and increased renal tubular cells apoptosis, indicating that LOCM is a feasible inducer for CI-AKI mice model. Taken together, we found that uninephrectomy (UPHT) combined with 24 h water deprivation and furosemide administration 20 min before LOCM (iohexol, 10 ml/kg) application is a feasible pretreatment to establish a novel CI-AKI mouse model.

## Introduction

Iodinated contrast media (ICM) are the most widely used drugs in diagnostic visualization technique in recent decades. However, ICM has side effects on kidney function [1–4], resulting in contrast-induced acute kidney injury (CI-AKI) which is a serious iatrogenic complication and may cause increased morbidity and mortality [5–7]. CI-AKI is the third leading cause of hospital-acquired acute kidney injury (AKI), accounting for about 12% of all AKI cases [8–10]. Three basic mechanisms of CI-AKI development involve hemodynamic effects, reactive oxygen species formation, and the direct cytotoxic effect caused by iodinated contrast media mainly targeting on renal tubular and vascular endothelial cells [11,12]. Considering the prolonged length of hospital stay, increased hospitalization cost, long admissions and poor outcome of CI-AKI [13] and complex and not fully understood pathophysiology of CI-AKI, it is urgent to discover and develop new therapeutic strategies against this disorder. Thus, an adequate and reproducible animal model, especially mouse model, is essential for CI-AKI study.

It has been reported that the nephrotoxicity of contrast medium is associated with its properties of osmolality, viscosity and direct chemotoxic effects. Accumulated studies indicated that low-osmolar contrast media (LOCM) was less nephrotoxic than high-osmolar contrast media (HOCM) and caused lower incidence of CI-AKI [14,15]. For that reason, LOCM, considered to be safer than the HOCM, is generally accepted and extensively used in clinical practice. Regrettably, most of the previous CI-AKI models especially mainly before 2014, with HOCM like urografin as the inducer, make modeling more available, but are seldom used for clinic application [16–18]. Those HOCM-induced AKI models cannot meet demands for frontier researches. So recently, New Zealand white rabbits and rats were widely used to make CI-AKI models with LCOM and different pretreatments [19–21]. Although gradually there are a few mice models with LCOM and confounding pretreatment such as ischemic damage to the kidneys or some nephrotoxic drugs [22–26], however, the research on CI-AKI model based on mice, especially male mice, with proper pretreatment similar to human risk factors was still blank, which was more suited to study-specific gene functions. Therefore, it is urgently needed to construct a reliable male mice model with proper pretreatment for the research on LOCM-induced AKI.

An ideal CI-AKI animal model should imitate the clinic pathogenesis process of patients with CI-AKI. The morbidity of CI-AKI greatly increased over 50% in intensive care unit (ICU) patients, much more than in normal ones [27]. Hypovolemia, micro-inflammation condition, or sepsis in ICU patients could cause functional damages to kidney [28]. In short, these preexisting kidney impairments remain clinically precipitating factors for the emerging of CI-AKI [12,29]. Consistent with these, it is hard to construct CI-AKI animal model with sheer contrast media treatment, and pretreatments before contrast media injection is required to cause functional damages to kidney. To date, the commonly used pretreatment methods are nephrotoxic drug, ischemic damage, dehydration such as water deprivation or furosemide injection, surgery such as UPHT or 5/6 nephrectomy [20,21,30]. Predisposing factors should be similar to human risk factors. Firstly, the ischemic damage and nephrotoxic drug can interfere with research results [31]. Secondly, the 5/6 nephrectomy are not so proper to test the protective role of the medicine due to more severe chronic kidney injury degree compared to UPHT. Thirdly, hypovolemia is the most important risk factors for the development of CI-AKI along with diabetes, prior CKD, hypotension and sepsis [12], so CI-AKI male mice model based on dehydration and UPHT is near the clinic setting and the complementary study to the rat model with the prior CKD risk factor [19].

In this study, considering that iohexol as a nonionic low-osmolar contrast media (CM) is widely used in hospitals [32], we chose it as a representative CM in our study and aimed to establish a novel reliable and reproducible LOCM-induced AKI model in male mouse comparable to the human contrast-induced nephropathy. Different dehydration treatments with UPHT were attempted to explore reliable preparatory conditions for CI-AKI mouse model construction, which were verified *in vivo* subsequently.

## Material and methods

### Chemicals and animals

The LOCM used in this study was iohexol (350 mg iodine/ml, 844 m Osm/kg of water and 10.4 cPs at 37 °C; GE Healthcare, Shanghai, China). Male C57BL/6 mice (18–20 g) were purchased from the Animal Center of Shanghai Jiaotong University, Shanghai, China. To increase the likelihood of AKI, The mice were acclimatized for 7 days before research and underwent UPHT 4 weeks before. The UPHT surgery was operated as previous study described [33]. All experimental protocols conformed to the Animal Care and Use Committee of Shanghai Jiaotong University School of Medicine and in compliance with the Guidelines for the Care and Use of Laboratory Animals (NIH Publication number 85-23, revised 1996).

### Ethics statement

All procedures involving these animals were approved by the Animal Protocol Committee of Shanghai Jiaotong University and conducted according to the Animal Care Committee at the Renji Hospital, School of Medicine, Shanghai Jiaotong University. The animal experiment ethics approval number is m20170324.

### In vivo experimental design

**Figure 1.**
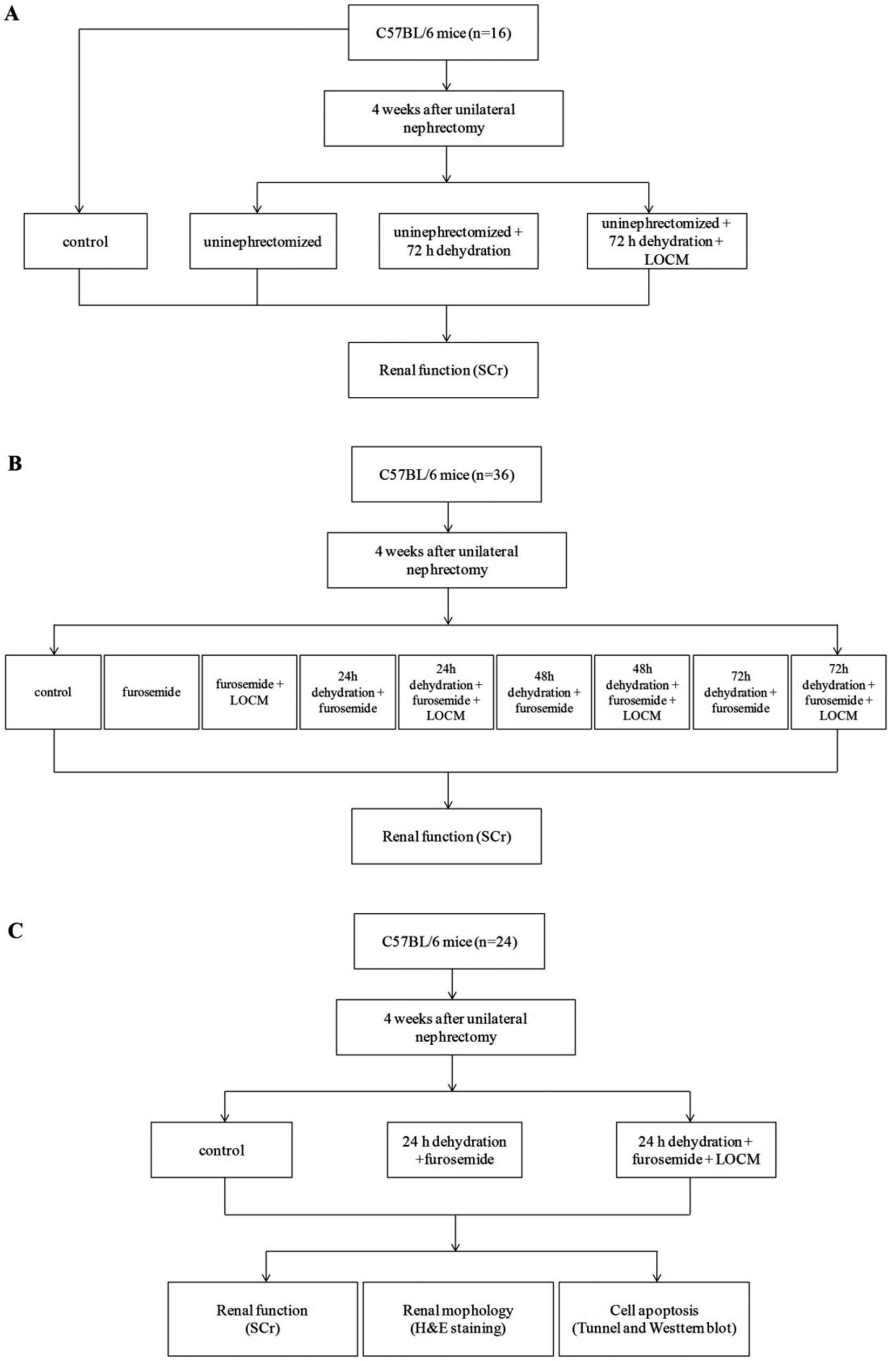
**Study protocol.** (A) Identify whether water deprivation + low-osmolar contrast media (LOCM) could induce contrast-induced acute kidney injury (CI-AKI) in mice. (B) Seek for the optimal precondition procedure. (C) Confirm the condition and analyze the toxic effects of LOCM on the kidney of mice.

Previous studies have documented contrast media injection solely in male mice is unable to induce AKI. All CI-AKI models require different kinds of pretreatments. The study was divided into three phases (Figure 1). The first phase includes multiple water deprivation pretreatment plans to find whether only water deprivation without furosemide combined with LOCM (iohexol, 10 mL/kg as previously described by other studies [21,23]) and UPHT can induce AKI in mice (Figure 1(A)). The length of water deprivation was according to previous documents [19]. The longest water deprivation time was 72 h. Thus, we chose UPHT + 72 h water deprivation as pretreatment. Sixteen male C57BL/6 mice were assigned to four groups randomly (4 mice/group): (1) control group: normal mice; (2) uninephrectomized group: mice underwent UPHT for four weeks; (3) uninephrectomized + 72 h water deprivation group: water deprivation for 72 h; (4) uninephrectomized + 72 h water deprivation + LOCM administration group: after water deprivation for 72 h, LOCM was injected *via* tail vein administration. Serum creatinine (SCr) concentrations were detected to evaluate kidney function.

According to our negative result of first phase and relevant research of furosemide, inhibiting tubular reabsorption and dilating renal blood vessels, as an efficient pretreatment in CI-AKI. Thus, we take furosemide into consideration and the second phase includes furosemide (10 mL/kg; Selleckchem, Shanghai, China) pretreatment combined with and without multiple water deprivation to find the optimal precondition procedure. Besides, to explore whether dehydration alone with water deprivation and furosemide but without LCOM after UPHT can cause kidney damage, so thirty-six uninephrectomized mice were randomly equational assigned to nine groups (4 mice/group) considering all the above purposes (Figure 1(B)): (1) control group: unilateral nephrectomized mice without any other treatment; (2) furosemide group: furosemide was injected *via* tail vein administration; (3) furosemide injection + LOCM administration group: furosemide was injected *via* tail vein administration 20 min before LOCM administration; (4) 24 h water deprivation + furosemide injection group: after water deprivation for 24 h, furosemide was injected *via* tail vein administration; (5) 24 h water deprivation + furosemide injection + LOCM administration group: after water deprivation for 24 h, furosemide was injected *via* tail vein administration, followed with LOCM administration 20 min later; (6) 48 h water deprivation + furosemide injection group: after water deprivation for 48 h, furosemide was injected *via* tail vein administration; (7) 48 h water deprivation + furosemide injection + LOCM administration group: after water deprivation for 48 h, furosemide was injected *via* tail vein administration 20 min before LOCM administration; (8) 72 h water deprivation + furosemide injection group: after water deprivation for 72 h, furosemide was injected *via* tail vein administration; (9) 72 h water deprivation + furosemide injection + LOCM administration group: after water deprivation for 72 h, furosemide was injected *via* tail vein administration, following by LOCM administration 20 min later.

According to our positive result of second phase, the third phase includes confirming the change of group 5 and analyzing the toxic effects on the kidney (Figure 1(C)). On the basis of phase one and two, another twenty-four mice were assigned into three groups (8 mice/group): (1) control group: unilateral nephrectomized mice; (2) 24 h water deprivation + furosemide injection group: after water deprivation for 24 h, furosemide was injected *via* tail vein. (3) 24 h water deprivation + furosemide injection + LOCM administration group: after water deprivation for 24 h, furosemide was injected *via* tail vein administration 20 min before LOCM administration.

The length in hours of water deprivation was approved by the ethics committee of Renji Hospital, School of Medicine, Shanghai Jiaotong University. All animals were bred in a climate-controlled room and had *ad libitum* access to food after injection until the study finished. In addition, all mice, except 2 mice in 72 h water deprivation + furosemide injection + LOCM administration group, were alive through the whole study. 24 h after all treatment in each group, blood was sampled from eyeball of mice after anesthesia, which make the mice comatose. We use cervical dislocation as an euthanasia method, then quickly cut the chest with cardiac perfusion involves intravenous administration of 50 mL PBS through the mice left atrial appendage, combined with kidney excision and renal capsule removing. Blood clotted for a minimum of 30 min, and then was centrifugated at 2000 g for 10 min to collect serum. The kidneys were also harvested for the future cell apoptosis detection and tissue staining assays.

### Mouse serum biomarkers investigation

Blood clotted for at least 30 min and was centrifugated at 2000 *g* for 10 min in order to collect serum. Serum creatinine (SCr) and blood urea nitrogen (BUN) concentrations were detected by Hitachi 7060 chemistry analyzer.

### Hematoxylin and eosin staining

The renal tissue was washed with 0.9% saline, fixed in 10% neutral buffered formalin and then embedded in 10% paraffin. Sections (5 μm thick) were stained with hematoxylin and eosin (H&E) for further analysis. Stained specimens were assessed by a pathologist with a light microscope (Leica DM 6000 B; Leica Microsystems, Wetzlar, Germany). We selected randomly 10 high-magnification (×400) fields of the cortex and outer stripe of the outer medulla to analysis the frequency and severity of renal lesions. The specimens were scored by the severity of foamy degeneration and detachment of tubular cells on a semi-quantitative scale: no injury (0), mild: < 25% (1), moderate: < 50% (2), severe: < 75% (3), and very severe: > 75% (4).

### dUTP Nick-End labeling assay

Terminal deoxynucleotidyl transferase-mediated deoxyuridin triphosphate nick end labeling (TUNEL) assay was conducted to measure cell apoptosis in renal tissues with an *in situ* cell death detection kit (Roche, Netley, NJ, USA). Brown labeled TUNEL positive cells were counted in 10 high-power (×400) fields.

### Western blotting

Mice tissues were lysed using a protein lysis buffer containing 20 mM Tris (pH 7.4), 1 mM EDTA, 1% Triton X-100, 150 mM NaCl, 1 mM EGTA, 2 mM sodium orthovanadateas aprotinin and 25 mM sodium pyrophosphate. Linearized proteins were separated using SDS-PAGE gel and transferred to polyvinylidene difluoride (PVDF) membranes (Roche, Netley, NJ, USA). The membranes were blocked with 5% skimmed milk in Tris-buffered saline and then incubated with primary anti-Caspase 3 (CST, Beverly, MA, USA), anti-Cleaved Caspase 3 (CST, Beverly, MA, USA), anti-Bcl-2 (Antibody Revolution, San Diego, CA, USA), anti-Bax (Abcam, Cambridge, MA, US), and anti-Tubulin (Proteintech, Chicago, IL, USA) antibodies. The samples were then incubated with horseradish peroxidase-conjugated anti-rabbit secondary antibody (R&D Systems China Co. Ltd, Shanghai, China). The bands were visualized using the ECL Western Blotting Kit (Biovision, Milpitas, CA, USA) and quantified using Image J software (NIH, USA).

### Statistical analysis

Statistical software SPSS Version 18.0 is used to perform statistical analysis. All data are presented as means ± SD. Student’s t-test test was performed to compare two groups. The means among more than two groups were evaluated by analysis of variance followed by Tukey’s multiple comparison. *p* < 0.05 was considered significant.

## Results

### Administration of water deprivation and LOCM was unable to induce AKI

**Figure 2.**
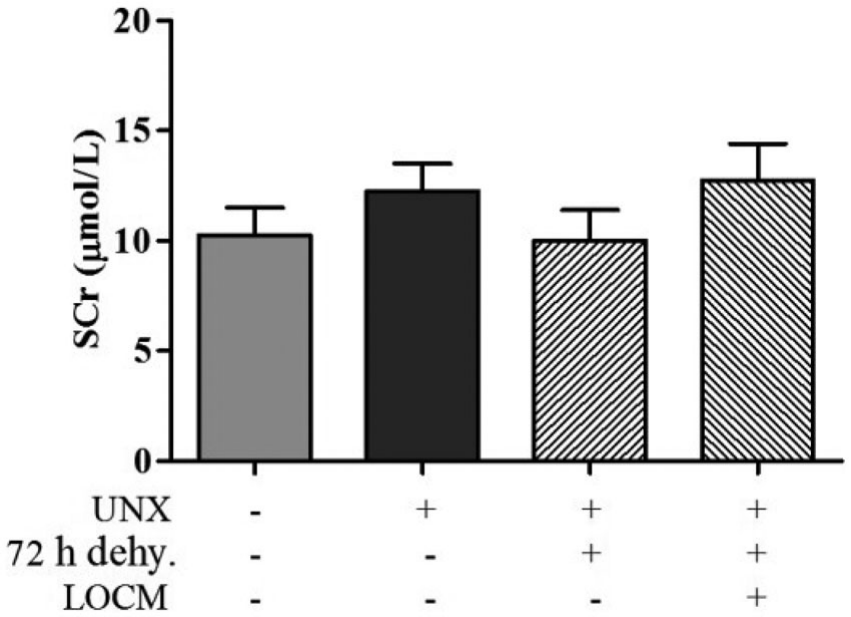
**Analysis of serum creatinine (SCr) levels based on the water and** **low-osmolar contrast media (LOCM)** **administration.** The SCr levels in conrol group (normal mice), uninephrectomy (unine.) group, uninephrectomized combined with 72 h water deprivation (dehy.) group (uninephrectomized mice treated with water deprivation for 72 h), uninephrectomized combined with 72 h water deprivation and LOCM injection group (uninephrectomized mice treated with water deprivation for 72 h, followed by LOCM administration).

No remarkably increased SCr level in mice was observed in mice after UPHT for 4 weeks. There was no difference in SCr levels between the normal group, uninephrectomized group, 72 h water deprivation group and 72 h water deprivation combined with LOCM administration group (Figure 2). These results suggested that merely combine of water deprivation and LOCM administration could not induce AKI in uninephrectomized mice and furosemide should be taken into account accordingly.

### Optimum conditions for inducing CI-AKI

**Figure 3.**
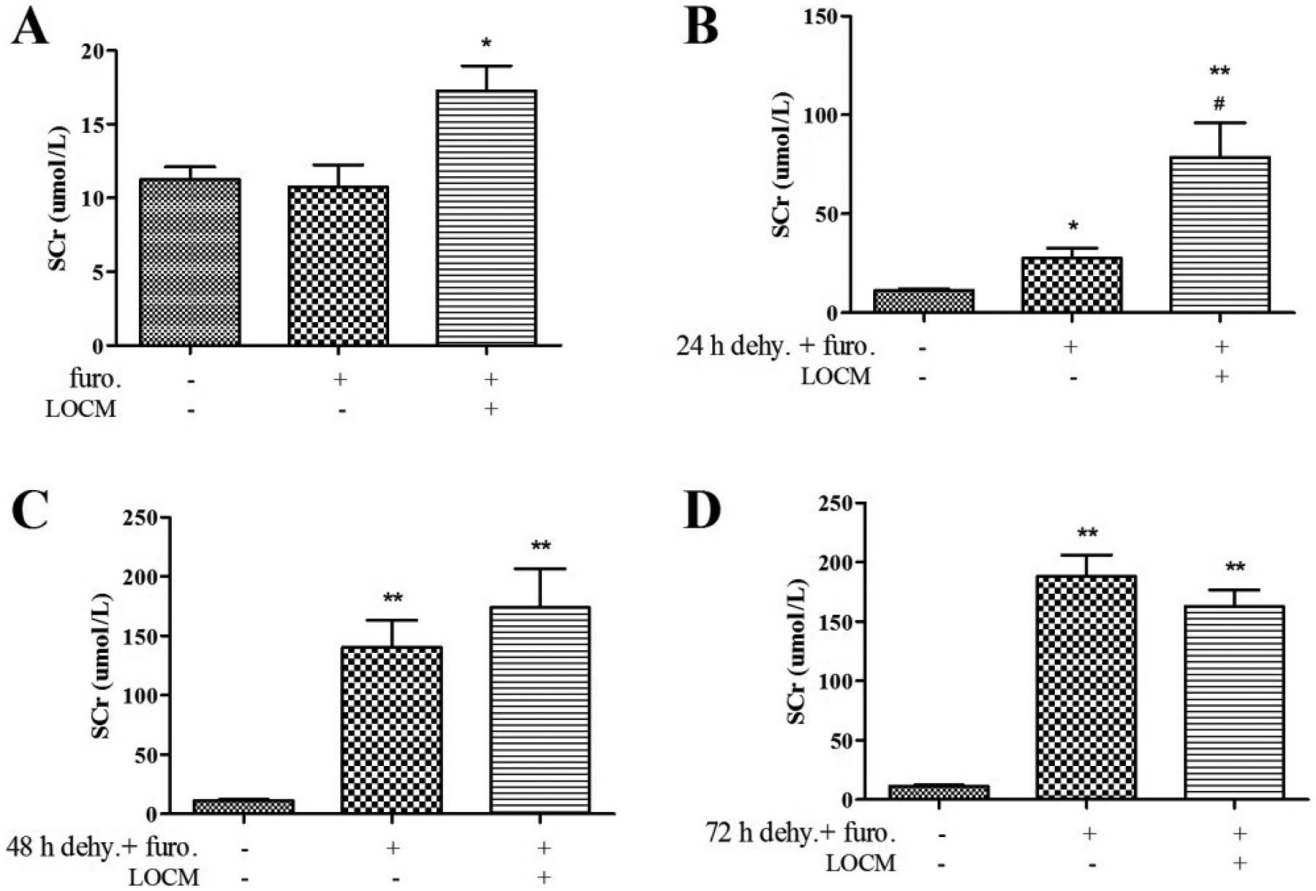
**24 h water deprivation combined with injection of furosemide and low-osmolar contrast media (LOCM) were optimized for inducing contrast-induced acute kidney injury (CI-AKI) in uninephrectomized mice.** (A) Changes in the level of serum creatinine (SCr) after injection of furosemide (furo.) with or without LOCM administration. *p < 0.05 vs. control. (B) Changes in the level of SCr after water deprivation (dehy.) for 24 h and furosemide injection with or without LOCM administration. *p < 0.05, **p < 0.01 vs. control; #*p* < 0.05 vs. furosemide injection only. (C) Changes in the level of SCr after water deprivation for 48 h and furosemide injection with or without LOCM administration. **p < 0.01 vs. control. (D) Changes in the level of SCr after water deprivation for 72 h and furosemide injection with or without LOCM administration. *p < 0.05, **p < 0.01 vs. control.

Since there were no statistical differences between the uninephrectomized mice and the normal mice in serum creatinine (SCr) levels, we chose uninephrectomized group as the control group in the following study. SCr levels of UPHT group (control group) are almost the same as that of UPHT + furosemide injection group (*p* > 0.05). Although the SCr levels were significantly increased in UPHT + furosemide injection + LOCM administration group compared to the control group (creatinine, 16.50 ± 1.29 μmol/L [furo. + LCOM] vs. 11.25 ± 0.96 μmol/L [control], *p* < 0.05; Figure 3(A)), it barely met the widely accepted definition of contrast-induced nephrology (CIN) characterized by a rise in SCr of more than 50%. Meanwhile, UPHT mice combine with water deprivation for 24 h and injection of furosemide, followed by LOCM injection or not showed statistic differences in SCr levels (creatinine, 21.50 ± 2.90 μmol/L [24h dehy.+furo] vs. 11.25 ± 0.96 μmol/L [control], *p* < 0.05; creatinine,69.50 ± 10.22 μmol/L [24h dehy.+furo.+LCOM] vs. 11.25 ± 0.96 μmol/L [control], *p* < 0.05; Figure 3(B)). While no statistical differences in SCr levels were detected between UPHT + 48 h water deprivation + furosemide injection group and UPHT + 48 h water deprivation + furosemide injection + LOCM administration group (*p* > 0.05) (Figure 3(C)), as well as between UPHT + 72 h water deprivation + furosemide injection group and UPHT + 72 h water deprivation + furosemide injection + LOCM administration group (*p* > 0.05) (Figure 3(D)) indicating LOCM was not the main cause of AKI in those groups. In short, these results indicated that water deprivation for 24 h combined with injection of furosemide is an optimal precondition procedure for inducing LOCM-induced AKI in unilateral nephrectomized male mice.

### LOCM results in severe kidney damage accompanied by cellular apoptosis

**Figure 4.**
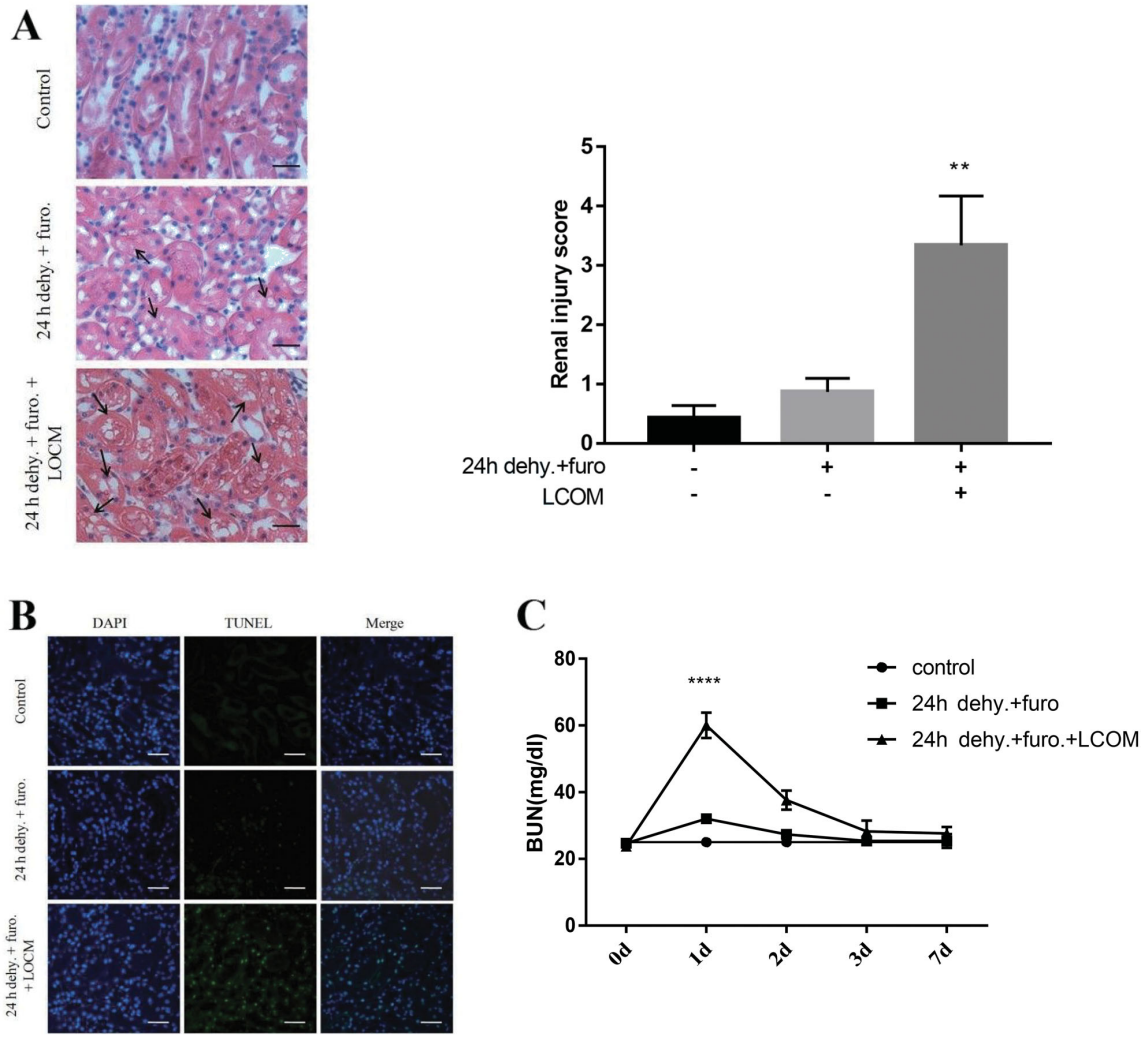
**Low-osmolar contrast media (LOCM) resulted in severe morphological injury and renal tubular cell apoptosis in uninephrectomized mice.** A. H&E staining and renal injury score of mouse kidney tissue sections. control, 4 weeks after uninephrectomy(UPHT) group; 24 h water deprivation (dehy.) + furosemide (furo.), uninephrectomized mice treated with water deprivation for 24 h, followed by furosemide injection; 24 h dehy. + furo. + LOCM group, uninephrectomized mice treated with water deprivation for 24 h, followed by furosemide injection and LOCM administration. Original magnification × 400; bar = 20 μm. Black arrows in show swelling and vacuolation in epithelial cells of the tubular. B. Representative kidney sections from mice were analyzed using TUNEL assay for apoptotic cell death. Mouse kidney cell apoptosis was monitored by TUNEL (Green). Nuclear morphological changes in renal cells were monitored with DAPI staining (Blue). Original magnification × 400; bar = 20 μm. C. Changes in the levels of blood urea nitrogen (BUN) at 1d, 2d, 3d, and 7d after the treatment, ****p < 0.0001 vs. control.

To access the effect of LOCM on kidney damage, mice were divided into 3 groups randomly: control group (unilateral nephrectomized mouse), 24 h water deprivation + furosemide injection group, as well as 24 h water deprivation + furosemide injection + LOCM administration group. As shown in Figure 4(A), there was slight injury of tubular cells in the 24 h water deprivation + furosemide injection group compared to the control group. Tubular degeneration with cell swelling and vacuolation in the 24 h water deprivation + furosemide injection + LOCM administration group was significantly severe compared with the control group and 24 h water deprivation + furosemide injection group. Consistent with these results, TUNEL staining showed that in 24 h water deprivation + furosemide injection + LOCM administration group, cell apoptosis was markedly increased compared with the control group and 24 h water deprivation + furosemide injection group (Figure 4(B)). Besides, to explore the question how long the AKI model can last, we assessed the time dependent BUN level. As Figure 4(C) shows, there were significant increases in BUN levels compared with the control group, which peaked at 24 h after injection (BUN, 60 ± 3.80 mg/dl [24h dehy. + furo. + LCOM] vs. 24.67 ± 0.58 mg/dl [control], *p* < 0.05; Figure 4(C)) and there were no differences in BUN levels between 24 h dehy. + furo. + LCOM group and control group at 72 h after injection.

**Figure 5.**
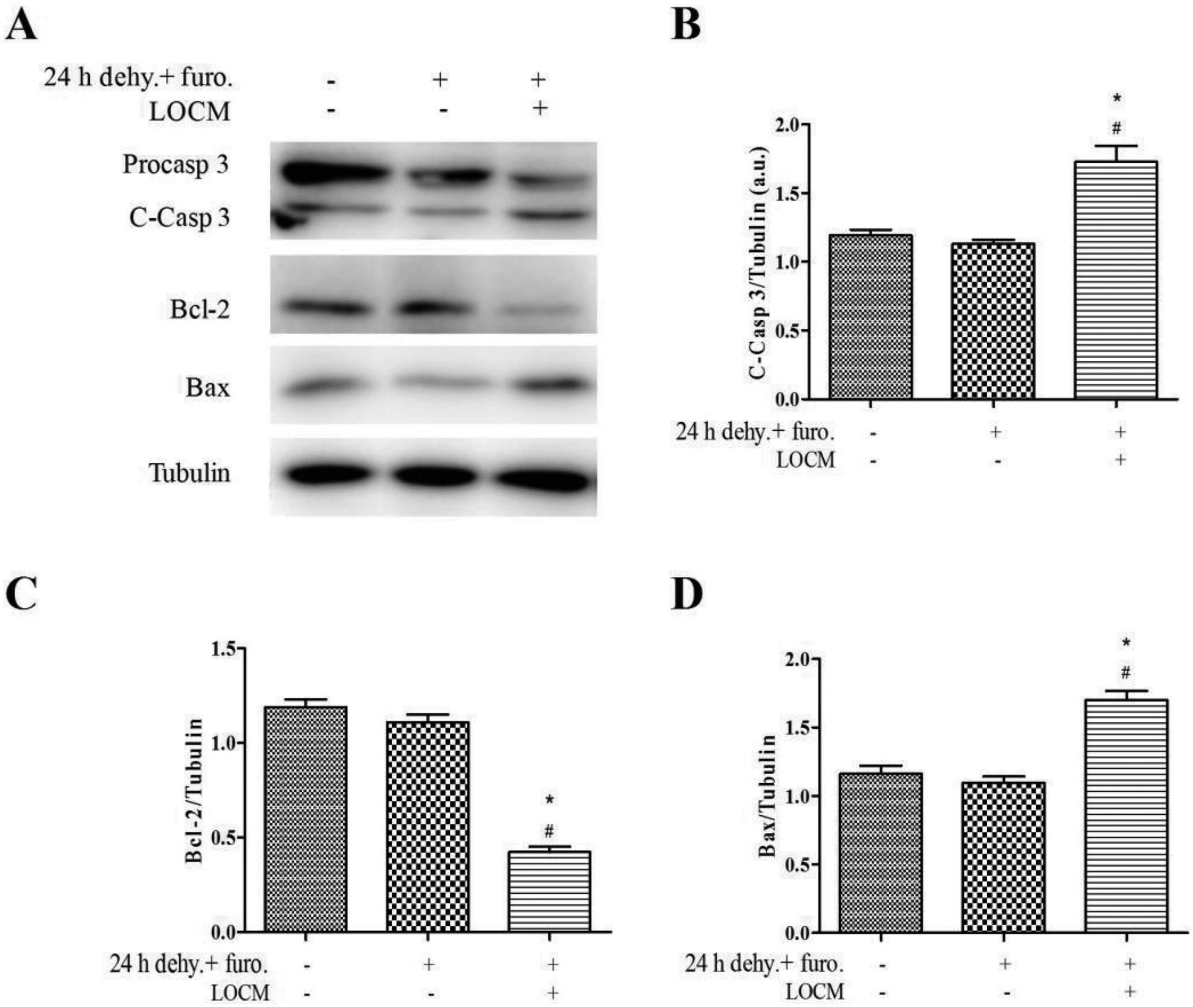
**Low-osmolar contrast media (LOCM) resulted in accumulations of apoptosis associated proteins in uninephrectomized mice.** (A) Western blotting analysis of apoptosis associated proteins in uninephrectomized mice, which were randomly assigned to three groups: control group (4 weeks after uninephrectomy(UPHT)), 24 h water deprivation (dehy.) + furosemide (furo.) group, as well as 24 h water deprivation + furosemide + LOCM group. (B-D) The gray intensity analysis of western blotting of cleaved caspase-3 (C- Casp 3), Bcl-2, and Bax. *p < 0.05 vs. control; #*p* < 0.05 vs. furosemide injection only.

Furthermore, 24 h water deprivation + furosemide injection + LOCM administration group showed significant cleaved caspase-3 (*p* < 0.05) and Bax (*p* < 0.05) up-regulation as well as Bcl-2 (*p* < 0.05) suppression compared with control group and 24 h water deprivation + furosemide injection group (Figure 5). These data indicated that LOCM induced severe renal injury, along with increased number of apoptotic cells in these novel mice models.

## Discussion

In animal experiments, it is a foundation and key of successful experimental study to establish a qualified animal model. Similarly, animal models are necessary to reveal the pathological changes in CI-AKI so as to prevent and treat this disease [34]. Different kinds of animals, especially rat which contains the advantages of convenient feeding, easy acquisition and genetic stability, were frequently applied to construct the CI-AKI models [16–18,35]. By contrast, CI-AKI models based on mice are relatively rare. Considering the higher drug consumption and longer growth period of large animals, small animals like mouse are proved to be a more ideal candidate for model establishment. Moreover, mice model is a preferred genetically engineered animal model for detecting the functions of target genes in transgenic or knockout mice, which would contribute to certifying the potential role of specific genes in therapeutic interventions prior to preclinical testing in humans [36]. Fortunately, a novel CI-AKI model was successfully built with C57BL/6 mouse as the background in our current study. Exposing healthy animal to contrast medium alone can rarely induce acute kidney injury [37] and by searching PubMed online articles, only one article about establishing a female mouse model of CIN by 10 gram of iodine per body weight (gI/kg) dose of iodixanol was found [23]. Thus, considering the sex difference [22,25], the pretreatments especially being more similar to the risk factors for human kidney damage compared to the interference of medicine such as indomethacin is still crucial to build a CI-AKI male mouse model. Multiple insults including UPHT, water deprivation, and furosemide injection [21], have been used to reduce kidney function, among which UPHT is an effective way to increase the burden of the residual kidney [30,38] and dehydration caused by water deprivation and furosemide is considered as being another appropriate method because of its accessibility and being comparable to hypovolemia risk factor of the human CIN [35,39]. In HOCM-induced AKI animal models, a prolonged water deprivation period (3 days) before HOCM injection in unilateral nephrectomized rats is sufficient to induce AKI ^[^^30^^]^. However, the LOCM possessed an osmolarity 2–3 times lower than HOCM. Thus, we speculate that sufficient pretreatments are required to perform LOCM-induced AKI mouse model.

In this study, we documented that solely administrating water deprivation failed to induce CI-AKI in UPHT mice. Multiple pretreatment plans involved water deprivation and furosemide administration were compared, based on which we found water deprivation for 24 h plus furosemide injection, followed by LOCM administration for 20 min, is an optimal method to induce CI-AKI in UPHT mice. Finally, we confirmed that LOCM successfully induced severe renal tubular injury and cell apoptosis by methods of H&E staining, TUNEL staining, and western blotting.

Comparing among multiple pretreatment plans, firstly, the slight but significant and large rises of SCr levels have been already achieved respectively in furosemide injection + LOCM administration group and 24 h water deprivation + furosemide injection + LOCM administration group compared to control group. Secondly, interestingly, severe dehydration alone with water deprivation and furosemide injection after UPHT can cause kidney damage when the deprivation time increased to more than 48 h, which means, in those groups, LOCM was not the main cause of AKI. Thirdly, most notably, the SCr levels in 24 h water deprivation + furosemide injection + LOCM administration group were over 3 times higher than 24 h water deprivation + furosemide injection group, which means it was LOCM that aggravated kidney injury on the basis of furosemide and 24 h water deprivation dramatically. Taken together, water deprivation for 24 h combined with furosemide injection and LOCM administration could be an efficient method to construct male mice model with CI-AKI.

When it comes to the reasons for the dose and type selection of iodinated contrast media and four weeks after the UPHT surgery as being the time selection, firstly, LOCM (iohexol, 10 mL/kg) is widely used in the previous study [21,23]. Although one study successfully adopted 15 mL/kg iohexol combined with dehydration plus furosemide (10 mL/kg) to establish CIN rat model [19], based on the principle of emphasizing the importance of minimizing the dose of CM in clinical practice, LOCM (iohexol, 10 mL/kg) combined with the proper pretreatment is enough to construct male mice model with CI-AKI. Besides, although iohexol is widely used in clinic practice, other common types, such as iodixanol^[^^32^^]^, need to be also further explored. At last, the time selection after the UPHT has a wide range of options between 2 and 4 weeks [30,33,40], mostly 3 weeks. The main aim is that the mice can recover from the surgery assault, so we choose 4 weeks for making sure their recovery, but we think 3 weeks selection is also ok as our previous team work described [41–44].

Severe renal morphologic damages were observed in the renal corticomedullary boundary zone. These findings in our study were similar to those findings in kidney biopsies from CI-AKI patients. The proximal tubules were mostly affected in our research as well as in the rats and clinically in patients.

The pathogenesis of CI-AKI is considered to be a paradigm of hypoxic/toxic injury, including altered renal microcirculation, hypoxia, and reactive oxygen species-mediated cellular injury [11,12,45]. LOCM agents possess high viscosity and osmolality which have vasoconstrictor effects and directly decreasing effect on the glomerular filtration rate. Thus, LOCM agents induce direct toxic effect on tubular epithelial cells markedly including tubular mitochondrial damage and oxidative stress damage as our previous team work showed [41–44]. In this study, the experimental mice were uninephrectomized, volume depleted and furosemide injected, so their residual kidneys were vulnerable for LOCM to induce severe kidney injury. The renal function of male mice with CI-AKI recovered about 3–7days after the treatment.

In conclusion, the male mice model based on uninephrectomy, 24 h water deprivation and furosemide administration is a reliable mouse model for LOCM-induced AKI, which shows some pathological symptoms similar to clinical CI-AKI. This mice model induced a significant reduction in renal function, severe renal tissue damage, and significant increased number of apoptotic tubular cells. For the first time, we established a LOCM-induced model based on C57BL/6 mice, which inspired further researches on contrast-induced nephropathy and might further provides benefits to patients suffering from acute kidney failure induced by contrast administration.
